# Colorectal endoscopic mucosal resection with submucosal injection of epinephrine versus hypertonic saline in patients taking antithrombotic agents: propensity-score-matching analysis

**DOI:** 10.1186/s12876-019-1114-x

**Published:** 2019-11-19

**Authors:** Daisuke Yamaguchi, Hisako Yoshida, Kei Ikeda, Yuki Takeuchi, Shota Yamashita, Amane Jubashi, Takahiro Yukimoto, Eri Takeshita, Wataru Yoshioka, Hiroko Fukuda, Naoyuki Tominaga, Nanae Tsuruoka, Tomohito Morisaki, Keisuke Ario, Seiji Tsunada, Kazuma Fujimoto

**Affiliations:** 1 Department of Gastroenterology, National Hospital Organization Ureshino Medical Center, Ureshino, Japan; 2 Department of Internal Medicine, Saga Medical School, Saga, Japan; 3 Department of Medical Statistics, Osaka City University Graduate School of Medicine, Osaka, Japan; 4 Faculty of Medicine, International University of Health and Welfare, Fukuoka, Japan

**Keywords:** Antiplatelet agents, Anticoagulants, Polypectomy, Bleeding, Perforation

## Abstract

**Background:**

Endoscopic mucosal resection (EMR) to remove colon polyps is increasingly common in patients taking antithrombotic agents. The safety of EMR with submucosal saline injection has not been clearly demonstrated in this population.

**Aims:**

The present study aimed to evaluate the efficacy and safety of submucosal injection of saline–epinephrine versus hypertonic saline in colorectal EMR of patients taking antithrombotic agents.

**Methods:**

This study enrolled 204 patients taking antithrombotic agents among 995 consecutive patients who underwent colonic EMR from April 2012 to March 2018 at Ureshino Medical Center. Patients were divided into two groups according to the injected solution: saline–epinephrine or hypertonic (10%) saline (*n* = 102 in each group). Treatment outcomes and adverse events were evaluated in each group and risk factors for immediate and post-EMR bleeding were investigated.

**Results:**

There were no differences between groups in patient or polyp characteristics. The main antithrombotic agents were low-dose aspirin, warfarin, and clopidogrel. Propensity-score matching created 80 matched pairs. Adjusted comparisons between groups showed similar en bloc resection rates (95.1% with saline–epinephrine vs. 98.0% with hypertonic saline). There were no significant differences in adverse events (immediate EMR bleeding, post-EMR bleeding, perforation, or mortality) between groups. Multivariate analyses revealed that polyp size over 10 mm was associated with an increased risk of immediate EMR bleeding (odds ratio 12.1, 95% confidence interval 2.0–74.0; *P* = 0.001).

**Conclusions:**

Two tested solutions in colorectal EMR were considered to be both safe and effective in patients taking antithrombotic agents.

## Background

Endoscopic polypectomy for colon lesions effectively reduces the risk of colorectal cancer [1, 2)]. Endoscopic mucosal resection (EMR), which involves the injection of fluid to expand the submucosal space, simplifies polypectomy and reduces the risk of adverse events [3–9)]. Post-polypectomy bleeding is the most common complication of endoscopic polypectomy, with reported incidences ranging from 0.65 to 8.6% [10–16)]. EMR with submucosal injection of epinephrine–saline or hypertonic saline solution enhances complete resection of lesions compared with simple polypectomy [17–20)]. Although both epinephrine solution and hypertonic saline have hemostatic effects that can prevent post-EMR bleeding, the efficacy of these two solutions in decreasing post-EMR bleeding in patients taking antithrombotic agents has not been clearly demonstrated [21–25)].

Antithrombotic agents, including antiplatelet agents and anticoagulants, are widely used to reduce the risk of thromboembolic events in patients with cerebro- and cardiovascular disease, deep vein thrombosis, and hypercoagulable status [26–29)]. Post-polypectomy bleeding after EMR is more commonly induced by anticoagulants than by antiplatelet agents [11, 30–32)]. Several clinical practice guidelines on gastrointestinal endoscopic procedures published in Europe, North America, Japan, and the Asia Pacific recommend that antithrombotic agents, especially aspirin, be continued during colonoscopic polypectomy. These clinical guidelines recommend that anticoagulants be discontinued during colorectal polypectomy in patients with low thromboembolic risks and be replaced with heparin for those with high thromboembolic risks [33–37)]. Several studies demonstrated that heparin replacement increased post-polypectomy and/or post-EMR bleeding compared with procedures without heparin or with original anticoagulants [28, 38, 39)].

Several solutions, including polidocanol, hyaluronic acid, and epinephrine–saline solution, have been used in colorectal EMR. However, the safety of these solutions has not been clearly demonstrated during EMR in patients taking antithrombotic agents [21–25)]. The aims of the present retrospective study were i) to compare the clinical outcomes of prophylactic injection of submucosal saline–epinephrine versus hypertonic saline for colorectal EMR in patients taking antithrombotic agents and ii) to identify the risk factors for immediate and post-EMR bleeding in these patients.

## Methods

### Patients

This retrospective chart review included 204 patients taking antithrombotic agents among 995 consecutive patients who underwent colonic EMR at the National Hospital Organization Ureshino Medical Center from April 2012 to March 2018. Patients >20 years of age who fulfilled the following criteria were candidates for the study: i) polyp diameter < 20 mm; ii) use of antithrombotic agents, including antiplatelet agents (low-dose aspirin, clopidogrel, ticlopidine, and cilostazol) and anticoagulants (warfarin and direct oral anticoagulants); and iii) normal coagulogram (platelet count: 140,000–379,000/μL, prothrombin time international ratio: 1.5–2.6). Patients with polyp diameter > 20 mm, abnormal coagulogram, and/or impaired normal blood clotting were excluded. Informed consent for the procedures was obtained from all patients who underwent colorectal EMR. The present study was conducted according to the Ethical Guidelines for Medical and Health Research Involving Human Subjects. The study protocol and the consent procedure were approved by the Ethics Review Committee of the National Hospital Organization Ureshino Medical Center (approval number 18–17).

### Procedure of EMR

**Fig. 1:**
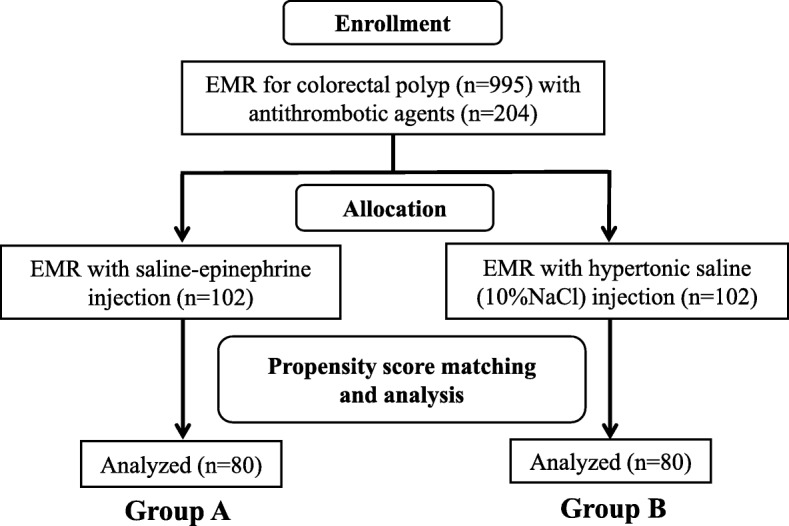
Flow diagram showing selection of patients taking antithrombotic agents who underwent colorectal endoscopic mucosal resection (EMR)

During the study period, 10 endoscopists with more than 3 years of experience in gastrointestinal colonoscopy performed colorectal EMR procedures using submucosal injection of either saline–epinephrine (0.01%) or hypertonic saline (10% NaCl), according to the judgment of the endoscopist. Patients were divided into two groups: the saline–epinephrine group (Group A) and the hypertonic saline group (Group B) (Fig. 1). The EMR procedure was performed with a colonoscope (PCF-Q260AZI; Olympus, Tokyo, Japan), snare (SnareMaster; Olympus), and electrosurgical unit (VIO 300, ERBE; Elektronedizin, Tübingen, Germany), with appropriate use of butylscopolamine or glucagon. Sedation was not used, except in patients with procedure-related pain or sedation request; in these patients, diazepam (5–10 mg), midazolam (0.05–0.1 mg/kg), pentazocine (15 mg), or pethidine hydrochloride (35 mg) was administered with monitoring of cardiorespiratory function [40].

**Fig. 2:**
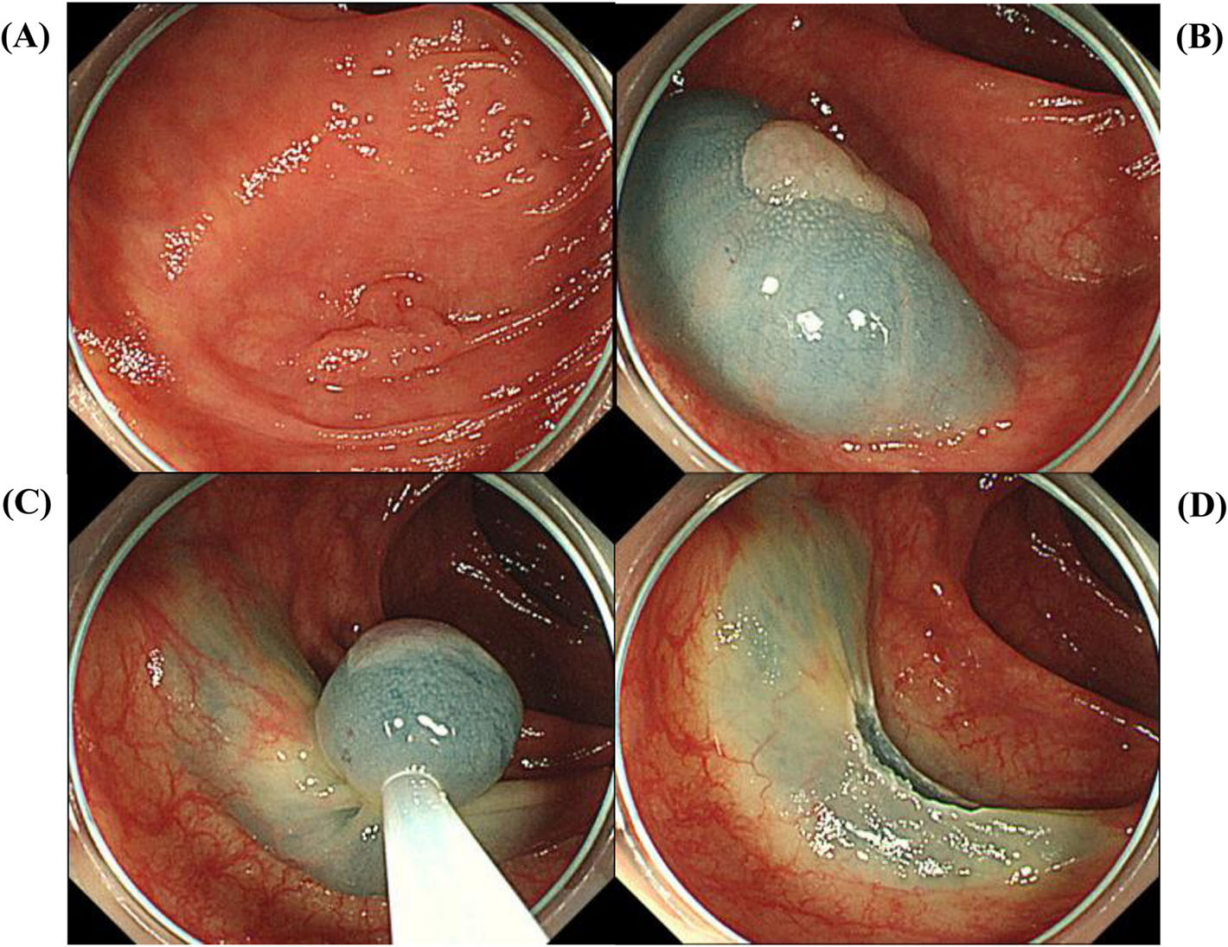
Colorectal endoscopic mucosal resection (EMR) with saline–epinephrine injection. **a** Sessile polyp in ascending colon, 10 mm in diameter. **b** Injection of saline–epinephrine for submucosal lifting. **c** Mucosal resection of polyp with the snare. **d** Post-EMR findings after en bloc resection

**Fig. 3:**
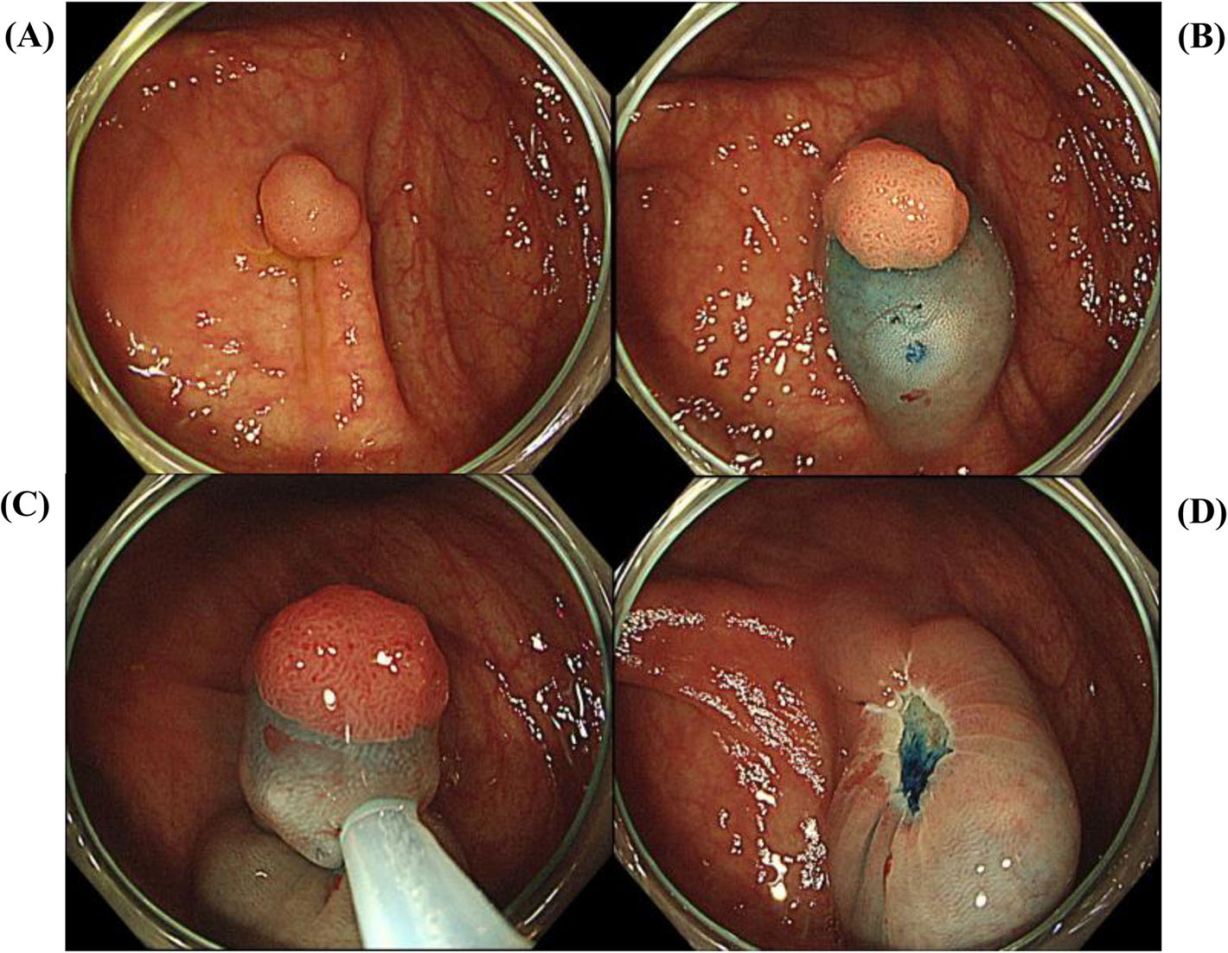
Colorectal endoscopic mucosal resection (EMR) with injection of hypertonic saline (10% NaCl). **a** Semi-pedunculated polyp in cecum, 8 mm in diameter. **b** Injection of hypertonic saline (10% NaCl) for submucosal lifting. **c** Mucosal resection of polyp with the snare. **d** Post-EMR findings after en bloc resection

After injection of saline–epinephrine (Fig. 2) or hypertonic saline (Fig. 3) into the submucosa with needle forceps to create an adequate mucosal bulge, the colorectal polyp was snared and resected [17, 22)]. Prophylactic clipping was performed in all patients. When bleeding was clinically suspected after EMR, emergency colonoscopy was performed to achieve hemostasis with hemoclips and/or electrocoagulation [19)].

### Clinical outcomes

Information on antithrombotic agents was recorded, including type, number, and management (continuation, cessation, or replacement) and comorbidity including Charlson comorbidity score was detected. Information on polyp lesions (size, location, and histological and macroscopic classifications), the endoscopist (specialist or trainee), and treatment outcomes (procedure time and en bloc resection rate) were reviewed after EMR. Specialist endoscopists were defined as those who had performed more than 40 EMR procedures over a period of at least 3 years after attaining the required fundamental skills and knowledge [41, 42)]. Immediate EMR bleeding was defined as hemorrhage during the procedure; post-EMR bleeding was defined as bleeding that occurred at least 1 h after the procedure. Adverse events of immediate EMR bleeding, post-EMR bleeding, perforation, and mortality within 30 days were recorded. Results are expressed as means ± standard deviations (SD).

The χ^2^ test was used to identify differences in the effectiveness rate between the two groups. Student’s t test was used for unpaired data to determine differences in means between the two groups. As indicated in Fig. 1, the two groups were compared after propensity-score matching. Propensity-score-matching analysis was used to control factors that might influence EMR treatment outcomes and adverse events. The two groups were matched in a 1:1 ratio (Group A, *n* = 80; Group B, *n* = 80) with propensity-score matching adjusted for five covariates (age, sex, anticoagulant agents, antiplatelet agents, and endoscopist) to minimize inherent bias (Table 5). This model yielded a c statistic of 0.67, indicating the ability to differentiate between Groups A and B. The caliper width of propensity-score matching was 0.05. Differences were considered statistically significant at *P* < 0.05. Univariate and multivariate logistic regression was performed to assess risk factors for immediate and post-EMR bleeding, with the explanatory variables of age, sex, anticoagulant agents, antiplatelet agents, multiple agents, heparin bridge therapy, polyp size, number of polyps, histological classification, endoscopist, and type of injection. All statistical analyses were performed with JMP version 13.0.0 (SAS Institute, Tokyo, Japan).

## Results

Among 995 consecutive patients who underwent colorectal EMR, 204 patients taking antithrombotic agents were included. Among these, 102 received saline–epinephrine injection and were allocated to Group A and 102 received hypertonic saline injection and were allocated to Group B (Fig. 1). There was no significant difference in baseline characteristics between the groups before propensity-score matching (Table 1). The median age of patients was 73.7 ± 8.7 years in Group A and 73.7 ± 8.3 years in Group B; 84 (82.4%) patients in each group were men. There were no significant differences between groups in the various comorbidities and Charlson comorbidity score. EMR procedures were performed by trainees more frequently in Group A than in Group B (72/102 vs. 50/102, respectively; *P* = 0.003).

**Table 1:** Characteristics of patients taking antithrombotic agents who underwent endoscopic mucosal resection of colorectal polyps with injection of epinephrine–saline (Group A) or hypertonic saline (Group B)

	Group A	Group B	*P* value
Number of patients (n)	102	102	
Age (years)	73.7 ± 8.7	73.7 ± 8.3	0.97
Sex, males	84 (82.4%)	84 (82.4%)	1.00
Alcohol drinking	39 (39.8%)	37 (37.7%)	0.88
Smoking	36 (36.7%)	33 (33.7%)	0.77
BMI (kg/m^2^)	23.5 ± 3.9	23.2 ± 3.6	0.57
History of colonoscopy	91 (89.2%)	91 (89.2%)	1.00
Using laxatives	22 (21.6%)	17 (16.8%)	0.48
Comorbidity			
Cardiovascular diseases	32 (31.3%)	27 (26.5%)	0.54
Cerebrovascular diseases	22 (21.6%)	27 (26.5%)	0.42
Chronic kidney diseases	11 (10.8%)	10 (9.8%)	1.00
Chronic liver damage	4 (3.9%)	2 (2.0%)	0.68
Diabetes mellitus	31 (30.4%)	35 (34.3%)	0.65
Hypertension	79 (77.5%)	84 (82.3%)	0.49
Malignant diseases	26 (25.5%)	23 (22.6%)	0.74
Charlson comorbidity score	2.2 ± 1.3	2.2 ± 1.4	1.00
Operators of procedure			
Trainees	72 (70.6%)	50 (49.0%)	0.003
Specialists	30 (29.4%)	52 (51.0%)	

Results are presented as mean ± SD or number of patients

Table 2 summarizes the types and management of antithrombotic agents. The main antithrombotic agents were low-dose aspirin, warfarin, and clopidogrel. The proportion of patients taking anticoagulant agents was significantly higher in Group B (39.2%) than in Group A (20.6%; *P* = 0.006). Before colorectal EMR, there was no significant difference between groups in the percentage of patients whose warfarin was replaced with heparin (14.7% in Group A vs. 23.5% in Group B) or with direct oral anticoagulants (1.0% in both groups). There was also no difference between groups in the percentage of patients whose cilostazol was replaced with other antiplatelet agents (14.7% vs. 10.8%).

**Table 2:** Types and management of antithrombotic agents in patients injected with epinephrine–saline (Group A) or hypertonic saline (Group B) for colorectal endoscopic mucosal resection (EMR)

	Group A	Group B	*P* value
Number of antithrombotic agents			
Single agent	88 (86.3%)	85 (83.3%)	0.70
Multiple agents	14 (13.7%)	17 (16.7%)	
Anticoagulants	21 (20.6%)	40 (39.2%)	0.006
Warfarin	12 (11.8%)	25 (24.5%)	
DOAC	9 (8.8%)	14 (13.7%)	
Management before EMR			
Cessation	5 (5.0%)	2 (2.0%)	0.45
Heparin replacement	15 (14.7%)	24 (23.5%)	0.15
DOAC replacement	1 (1.0%)	1 (1.0%)	1.00
Antiplatelet agents	85 (83.3%)	69 (67.6%)	0.014
Aspirin	36 (35.3%)	40 (39.2%)	
Clopidogrel	20 (19.6%)	12 (11.8%)	
Ticlopidine	6 (5.8%)	1 (1.0%)	
Cilostazol	16 (15.7%)	9 (8.8%)	
Others	7 (6.9%)	7 (6.9%)	
Management before treatment			
Cessation	4 (3.9%)	3 (2.9%)	1.00
Cilostazol replacement	15 (14.7%)	11 (10.8%)	0.53

Results are presented as mean ± SD or number of patients. DOAC: direct oral anticoagulants

As shown in Table 3, the colonic polyp characteristics were not different between the groups. The average number of tumors was 2.6 ± 1.9 in Group A and 2.9 ± 2.6 in Group B. The average tumor size was 10.0 ± 5.8 mm and 10.0 ± 5.0 mm. Regarding polyp location, sigmoid polyps were most common in both groups (34.3% vs. 33.3%). Macroscopically, the 0–Is type was most common in both groups (32.3% vs. 36.3%); adenoma was the most common histological classification (78.4% in both groups).

**Table 3:** Characteristics of colorectal polyps treated with endoscopic mucosal resection in Group A (epinephrine–saline injection) and Group B (hypertonic saline injection)

	Group A	Group B	*P* value
Number of polyps (n)	2.6 ± 1.9	2.9 ± 2.6	0.35
Size of polyps (mm)	10.0 ± 5.8	10.0 ± 5.0	0.94
Location of polyps			0.16
Cecum	2 (2.0%)	4 (3.9%)	
Accending	25 (24.5%)	27 (26.5%)	
Transverse	17 (16.6%)	26 (25.5%)	
Descending	13 (12.8%)	4 (3.9%)	
Sigmoid	35 (34.3%)	34 (33.3%)	
Rectum	10 (9.8%)	7 (6.9%)	
Macroscopic classification			0.31
0-Is	33 (32.3%)	37 (36.3%)	
0-Ip	20 (19.6%)	19 (18.6%)	
0-Isp	33 (32.4%)	21 (20.6%)	
0-IIa	7 (6.9%)	14 (13.7%)	
0-IIc	2 (2.0%)	4 (3.9%)	
Laterally spreading tumor	7 (6.9%)	7 (6.9%)	
Histological classification			0.49
Adenoma	80 (78.4%)	80 (78.4%)	
Adenocarcinoma	18 (17.7%)	14 (13.7%)	
Hyperplastic polyp	3 (2.9%)	4 (3.9%)	
Others	1 (1.0%)	4 (3.9%)	

Results are presented as mean ± SD or number of patients

Propensity-score matching created 80 matched pairs in the present study. As shown in Table 4, before propensity-score matching, significant differences were present in the proportion of patients taking anticoagulant agents (20.6% in Group A vs. 39.2% in Group B, *P* = 0.006) and of trainee endoscopists (70.6% vs. 49.0%, *P* = 0.003). Propensity-score matching averaged the differences in five covariates. Table 5 shows EMR treatment outcomes and adverse events after propensity-score matching. Procedure time was similar in both groups (29.5 ± 19.5 s vs. 31.0 ± 18.8 s, *P* = 0.65). The percentage of patients who underwent en bloc resection was not different between groups (95.0% vs. 97.5%, *P* = 0.68). Regarding adverse events, there were no significant differences in the incidence of immediate EMR bleeding (7.5% vs. 2.5%, *P* = 0.28), post-EMR bleeding (8.8% vs. 3.8%, *P* = 0.33), time to post-EMR bleeding (1.7 ± 1.3 days vs. 2.3 ± 1.5 days, *P* = 0.58), incidence of perforation (0.0% in both groups, *P* = 1.00), or mortality rate (0.0% in both groups, *P* = 1.00). No cerebrovascular events occurred during or after EMR procedures in the present study. All patients with immediate EMR bleeding or post-EMR bleeding were successfully treated with endoscopic hemostasis.

**Table 4:** Characteristics of patients before and after propensity-score matching in Group A (epinephrine–saline injection) and Group B (hypertonic saline injection)

	Before propensity score matching			
	Group A	Group B	*P* value	Standardized difference
Number of patients (n)	102	102		
Age (years)	73.7 ± 8.7	73.7 ± 8.3	0.97	0.02
Sex, male	84 (82.4%)	84 (82.3%)	1.00	0.00
Anticoagulants	21 (20.6%)	40 (39.2%)	0.006	0.42
Antiplatelet agents	85 (83.3%)	69 (67.6%)	0.014	0.37
Operators of trainees	72 (70.6%)	50 (49.0%)	0.003	0.45
After propensity score matching				
Number of patients (n)	80	80		
Age (years)	73.2 ± 8.5	73.4 ± 8.3	0.87	0.07
Sex, male	66 (82.5%)	66 (82.5%)	1.00	0.00
Anticoagulant agents	18 (22.5%)	20 (25.0%)	0.85	0.06
Antiplatelet agents	63 (78.8%)	64 (80.0%)	1.00	0.03
Operators of trainees	50 (62.5%)	48 (60.0%)	0.87	0.05

Results are presented as mean ± SD or number of patients

**Table 5:** Treatment outcomes and adverse events of colorectal endoscopic mucosal resection (EMR) after propensity-score matching in Group A (epinephrine–saline injection) and Group B (hypertonic saline injection)

	Group A	Group B	*P* value
Procedure time (min)	29.5 ± 19.5	31.0 ± 18.8	0.65
En bloc resection	76 (95.0%)	78 (97.5%)	0.68
Adverse events			
Immediate EMR bleeding	6 (7.5%)	2 (2.5%)	0.28
Post EMR bleeding	7 (8.8%)	3 (3.8%)	0.33
Time to post EMR bleeding (days)	1.7 ± 1.3	2.3 ± 1.5	0.58
Perforation	0 (0%)	0 (0%)	1.00
Mortality	0 (0%)	0 (0%)	1.00

Results are presented as mean ± SD or number of patients

Table 6 lists the risk factors for immediate and post-EMR bleeding among patients taking antithrombotic agents. Only polyp size greater than 10 mm increased the risk of immediate EMR bleeding in univariate analysis (odds ratio, 5.57; 95% confidence interval [CI], 1.27–24.5; *P* = 0.024) and in multivariate analysis (odds ratio, 12.1; 95% CI, 2.0–74.0; *P* = 0.001). No risk factor for post-EMR bleeding was detected in the present study. The use of saline–epinephrine versus hypertonic solution for injection was not related to the risk of bleeding during or after colorectal EMR.

**Table 6:** Univariate and multivariate analysis of risk factors for bleeding during and after colorectal endoscopic mucosal resection (EMR)

	Risk factor for immediate EMR bleeding				Risk factor for post EMR bleeding			
Variables	Odds ratio	95% CI		*P* value	Odds ratio	95% CI		*P* value
Univariate analysis								
Age, >75 y	0.48	0.09	2.48	0.48	2.38	0.64	8.79	0.20
Sex, male	3.05	0.39	13.5	0.15	0.51	0.06	4.16	1.00
Anticoagulant agents	1.07	0.20	5.56	1.00	2.27	0.61	8.53	0.25
Antiplatelet agents	0.77	0.15	3.99	0.67	0.58	0.14	2.39	0.43
Multiple agents	0.84	0.10	7.20	1.00	1.54	0.30	7.73	0.64
Heparin replacement	1.07	0.21	5.56	1.00	0.79	0.16	3.90	1.00
Size of polyps, >10 mm	5.57	1.27	24.5	0.024	2.11	0.56	7.90	0.27
Number of polyps, > 2	0.23	0.03	1.93	0.26	2.74	0.74	10.2	0.17
Histological classification, 0-Ip	3.81	0.74	19.4	0.14	1.21	0.34	4.34	1.00
Operator of trainees	4.69	0.56	39.1	0.15	0.95	0.26	3.49	1.00
Injection of epinephrine	3.16	0.62	16.2	0.28	2.46	0.61	9.88	0.33
Multivariate analysis								
Size of polyps, >10 mm	12.1	2.00	74.0	0.001				

95% CI: 95% confidence interval

## Discussion

EMR is a standard procedure associated with substantial adverse events in the treatment of gastrointestinal lesions. Bleeding is the most common adverse event of colorectal EMR [11–17)]. Submucosal injection of epinephrine–saline solution, which is an effective method for colorectal EMR, especially in flat or sessile lesions, is widely used because of its simplicity, low cost, and wide availability [21)]. Hypertonic saline injection, which creates a relatively longer-lasting submucosal cushion because of its viscosity, enables EMR without apparent tissue damage [25)].

In the present study, both epinephrine–saline solution and hypertonic saline solution were effective for EMR in patients taking antithrombotic agents. Treatment outcomes (procedure time and rate of en bloc resection) were similar for EMR with both solutions. Regarding adverse events, no perforation or fatality related to EMR was observed in the present study; the incidence of immediate EMR bleeding (7.5% in Group A vs. 2.5% in Group B), post-EMR bleeding (8.8% vs. 3.8%), and time to post-EMR bleeding (1.7 ± 1.3 days vs. 2.3 ± 1.5 days) did not differ with injection of epinephrine–saline solution versus hypertonic saline solution. All bleeding resolved with endoscopic hemostatic methods, including high-frequency soft coagulation and/or hemoclip.

Several previous studies have reported bleeding rates of 0.65 to 8.6% after simple colorectal polypectomy with or without antithrombotic agents [10–16)]. The reported rate of post-colorectal EMR bleeding is 9.3 to 26.3% in previous studies on the use of antithrombotic agents [18, 28, 29, 43)]. The results in the present study regarding bleeding after EMR in patients taking antithrombotic agents are consistent with these previous studies and indicate the safety of colorectal EMR with injection of epinephrine–saline or hypertonic saline in patients taking antithrombotic agents. Lesion size over 10 mm was a risk factor for immediate and post-EMR bleeding in multivariate analysis in the present study. The type of solution used for injection in the EMR procedure was not a risk factor for bleeding, indicating that both epinephrine–saline and hypertonic saline can be used for colorectal EMR.

The present retrospective chart review had several limitations. The type of antithrombotic agent taken and the skill of the colorectal EMR endoscopist differed between groups. Submucosal injection of epinephrine-saline or hypertonic saline for EMR was selected by the endoscopist in the present study, and the trainee tended to use the solution of epinephrine-saline because of speculated advantage for prevention of bleeding of EMR. Propensity-score matching was used for statistical analysis to reduce the bias between groups including the endoscopist’s bias [44)]. The present study did not include polyps larger than 20 mm, although lesion size was a risk factor for bleeding during EMR.

## Conclusions

Colonic EMR procedures with the two tested solutions in the present study were safe and effective in patients taking antithrombotic agents as there were no serious complications with submucosal injection of epinephrine–saline or hypertonic saline.

## Data Availability

The datasets used and/or analyzed during the current study are available from the corresponding author on reasonable request.

## Abbreviations

CI: Confidence interval
EMR: Endoscopic mucosal resection
OR: Odds ratio
SD: Standard deviation
